# Effects of Acupuncture on Alzheimer's Disease in Animal-Based Research

**DOI:** 10.1155/2017/6512520

**Published:** 2017-09-27

**Authors:** Sunjung Park, Jun-Hwan Lee, Eun Jin Yang

**Affiliations:** ^1^Department of Clinical Research, Korea Institute of Oriental Medicine, 1672 Yuseong-daero, Yuseong-gu, Daejeon 305-811, Republic of Korea; ^2^Korean Medicine Life Science, University of Science and Technology (UST), Daejeon 34054, Republic of Korea

## Abstract

Alzheimer's disease (AD) is a chronic neurodegenerative disease characterized by the accumulation of amyloid beta (A*β*) plaques, neurofibrillary tangles, and severe functional deficits in the brain. The pathogenesis and treatment of AD remain topics of investigation and significant global socioeconomic issues. The effect of complementary medicine has been investigated in managing AD. Acupuncture, a form of therapy practiced for more than 3000 years, has shown positive effects on several neurological disorders including AD. Animal studies have evaluated the specific utility and neuropathological mechanisms addressed by acupoint manipulation; however, no study has summarized the relationships among different acupoints and their therapeutic effects in the context of AD. Therefore, we reviewed the effects of acupuncture at different acupoints in animal models of AD. In general, acupuncture produced therapeutic benefits in rodent models of AD. Studies demonstrate the utility of GV20 as a valuable acupoint for electroacupuncture and manual acupuncture. GV20 stimulation suppresses A*β* generation, improves glucose metabolism, and attenuates neuropathological features in various disease models. However, a lack of sufficient evidence in preclinical and clinical studies makes these results controversial. Additional studies are required to confirm the exact utility of specific acupoints in clinically managing AD.

## 1. Introduction

AD is a neurodegenerative disorder that is clinically characterized by progressive memory loss and cognitive deficits [1]. AD is the fourth leading cause of death in individuals over 65 years of age worldwide and is the underlying cause of 60–70% of cases of dementia. The global prevalence of dementia was estimated to be 5.3 million in 2015, with potential for this number to approach 80 million by 2040. Neuropathological hallmarks of AD include the abnormal production and accumulation of amyloid beta (A*β*) plaques and neurofibrillary tangles of hyperphosphorylated tau protein in the brain [2]. Accordingly, the uncontrolled generation of pathological A*β* and tau hyperphosphorylation is thought to drive neuronal loss and cognitive impairment in AD. Despite global awareness about the severity and socioeconomic effects of AD, effective treatments remain an important unmet need. Treatments based on Western medical science are proposed to delay functional memory impairment but produce unreliable effects and only delay disease progression in best-case scenarios [3].

Acupuncture is a traditional therapy that has been practiced in Korea, China, and Japan for centuries and is considered a useful form of complementary medicine [4, 5]. The efficacy of acupuncture has been demonstrated for treating numerous of severe disease states, including gastrointestinal disorders [6], breast cancer [7], colorectal cancer [8], chronic pain [9], and cognitive impairment. Common acupuncture techniques include electroacupuncture (EA) and manual acupuncture (MA). MA involves the insertion of fine stainless steel needles into specific acupoints, followed by manual manipulation such as twisting or thrusting [10]. EA is a technique in which two needles are inserted to generate electric current [10] and is generally a more common technique than MA. An important advantage of EA is that it combines the beneficial effects of transcutaneous electric nerve stimulation and MA [3].

EA and MA have demonstrated therapeutic utility for the treatment of AD in numerous animal studies [13–24]. For example, EA stimulation at GV14 and BL23 downregulates beta-secretase 1 (BACE1), an enzyme responsible for A*β* generation in AD, and increases ATP levels in the hippocampus of AD mice [14]. MA stimulation at ST36 alleviates glycerol metabolism in a rat model of AD generated by D-galactose injection [22]. Studies suggest that acupuncture has positive effects on cognition in AD and dementia by modulating neuronal signaling pathways. These pathways [13, 17, 19, 20, 22, 24] include those related to apoptosis, cell survival, and glucose metabolism and are suggested to mediate the beneficial effects of acupuncture on cognitive and physiological functions in animal models. Moreover, studies suggest that specific acupoints may exert specific therapeutic effects.

While several reviews have summarized data gleaned from studies in animal models of AD, no previous review has summarized the relationships between acupuncture stimulation at specific acupoints and their therapeutic effects in AD. Here, we review recent animal studies supporting the use of acupuncture as an effective therapeutic tool in AD and highlight specific acupoints that have demonstrated utility for targeting the neuropathological mechanisms of AD.

## 2. Effects of Acupuncture on AD

### 2.1. Electroacupuncture

**Figure 1:**
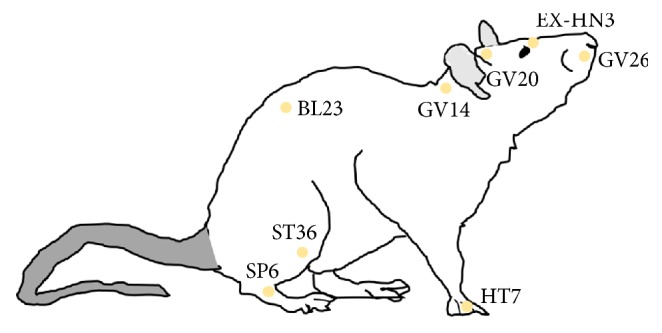
*Standard acupuncture acupoint locations in mouse and rat*. GV26 is located at the junction of the upper and middle third of the philtrum. GV20 is located on the midline between the top of the ears. GV14 is located below the spinous process of the seventh vertebrae at the approximate level of the shoulders. BL23 is located on the posterior midline at the level of the lower border of the spinous process of the second lumbar vertebra. HT7 is located on the wrist at the ulnar end of the transverse crease of the wrist, in the depression on the radial side of the tendon musculus flexor carpi ulnaris. ST36 is located below the lower border of the patella, one finger width lateral from the anterior border of the tibia. SP6 is located on the inside of the leg just above the ankle. EX-HN3 is located on the anterior midline, between the eyes. Anatomical locations of the stimulated acupuncture points in mice and rats were equivalent to the acupoints in humans [11, 12]. The location of EX-HN3 was determined in accordance with the National Acupuncture Society for Experimental Research “Laboratory Animal Acupuncture Atlas.”

EA has been widely used in modern investigations of acupuncture because it can be standardized in terms of frequency, voltage, and duration [3]. Several studies have suggested that EA stimulation produces cognitive improvements and positive changes in AD-related pathology in rodent disease models [14–18, 20, 21, 23, 24] For EA, acupoints GV14 (Daechu) and BL23 (Sinsu) are the most well studied in animal models of AD (Figure 1). Multipoint EA stimulation at GV14 and BL23 significantly decreases hippocampal A*β* accumulation in senescence accelerated mouse-prone8 (SAMP8) transgenic mice [14], which is an AD-like model characterized by cortical and hippocampal A*β* accumulation [13, 14]. Another important protein implicated in AD is 5′ adenosine monophosphate-activated protein kinase (AMPK), which serves as a master regulator of cellular energy homeostasis and thus glucose and lipid metabolism [14]. Activation of AMPK represses tau phosphorylation and A*β* production. The phosphorylation-dependent activation of AMPK is increased in SAMP8 mice after EA at GV14 and BL23 [14], suggesting that EA at these acupoints may affect glucose metabolism and ATP synthesis in the AD brain. Consistent with this suggestion, EA stimulation at GV14 and BL23 increases cortical and hippocampal ATP levels in SAMP8 mice. Cortical and hippocampal Sirtuin1 (SIRT1) and peroxisome proliferator-activated receptor *γ* coactivator 1-*α* (PGC1-*α*) were also upregulated and PGC1-*α* acetylation was decreased in SAMP8 mice after GV14 and BL23 EA stimulation [15]. In another study by the same group, EA at GV14 and BL23 reduced A*β* generation and downregulated BACE1 in the hippocampus of SAMP8 mice, which was associated with improvements in spatial learning and memory (Table 1) [16]. In summary, concurrent stimulation of GV14 and BL23 may regulate glucose metabolism, ATP synthesis, and A*β* generation to improve AD-like symptoms in SAMP8 mice.

GV20 (Baekhoe) has been used for single acupoint therapy in animal models of AD (Figure 1). EA stimulation at GV20 in APP/PS1 transgenic mice expressing a chimeric mouse/human amyloid precursor protein (APP) and a mutant human presenilin 1 (PS1) significantly ameliorated learning and memory deficits [20, 21]. Studies suggests that brain-derived neurotrophic factor (BDNF) is a critical factor in adult neurogenesis and memory [26, 27], which is deficient in patients with AD [28]. GV20 stimulation was found to upregulate BDNF in the hippocampus and cortex of APP/PS1 transgenic mice [20, 21]. Moreover, mature BDNF and pro-BDNF expression, the BDNF/pro-BDNF ratio, and tropomyosin receptor kinase (Trk) phosphorylation were enhanced by EA at GV20 in APP/PS1 mice [21]. Furthermore, it was found that GV20 stimulation increased neurogenesis [20] and decreased neuronal apoptosis [21] in the APP/PS1 mouse brain. Taken together, it can be hypothesized that GV20 promotes BDNF signaling to enhance neurogenesis and decrease cell death in the APP/PS1 brain. EA stimulation at GV20 has also been reported to affect glial fibrillary acidic protein (GFAP) and N-myc downstream-regulated gene 2 (NDRG2) signaling in the APP/PS1 mouse brain; GFAP and NDRG2 upregulation and associated memory deficits were significantly improved after EA at GV20 (Table 1) [23].

GV20 has also been studied in the context of multipoint EA stimulation. EA at GV20 and BL23 ameliorated memory impairment in a rat model of AD induced by A*β*_1-40_ injection. EA at GV20 and BL23 was found to reverse A*β*-induced downregulation of B-cell lymphoma 2 (Bcl-2), to upregulate Bcl-2 associated X protein (BAX) expression, and to upregulate Notch in a rat model of AD [17]. In another study, EA stimulation at GV20 and BL23 significantly improved cognitive impairment and reduced the brain expression of A*β* and p-tau proteins [24].

In the A*β*_1-40_ injection model of AD, EA at GV20 and BL23 restored peroxisome proliferator-activated receptor gamma (PPAR-*γ*) expression and mitigated increases in phosphorylated-p38 mitogen-activated protein kinase (p-p38MAPK) [24]. Taken together, these findings suggest that EA stimulation at GV20 and BL23 improves cognitive deficits via inhibition of the Notch pathway [17] and/or upregulation of PPAR-*γ* [24] in rat models of AD (Table 1).

Finally, Jiang and colleagues examined the utility of multipoint EA stimulation of GV20, GV26 (Sugu), and EX-HN3 (Yintang) in SAMP8 mice (Figure 1) and determined that stimulation improved hippocampal glucose metabolism [18], consistent with the results of studies examining single-point EA of GV20. Multipoint EA at GV20, GV26, and EX-HN3 also improved spatial learning and memory in SAMP mice. These findings ultimately highlight the importance of metabolic changes in AD and the potential for GV20 EA to promote healthy glucose and energy metabolism in the aging or pathological AD brain (Table 1).

### 2.2. Manual Acupuncture

MA is widely used as a traditional therapy and involves twisting, thrusting, or other manipulations of the acupuncture needle as a key feature. Several studies have demonstrated the utility of MA for improving cognitive impairments in animal models of AD. GV20 has been identified as a valuable acupoint for MA as well as EA. MA at GV20 improves memory impairment in a scopolamine-induced rat model of AD [19]. This benefit was associated with increases in choline acetyltransferase (ChAT), BDNF, and cAMP response element binding (CREB) expression in the hippocampus [19]. Decreased cholinergic function in the brain was also observed in patients with AD, producing defects in memory and cognitive function. Cholinergic function is associated with attention and working memory [29]. MA at GV20 was also found to upregulate hippocampal choline transporter 1 (CHT1), vesicular acetylcholine transporter (VAChT), BDNF, and CREB in AD model rats (Table 1) [19], suggesting that manual GV20 stimulation may promote cholinergic neurotransmission as a component of its effects on memory and cognition.

In a previous study, MA at ST36 and SP6 was found to modulate the function of hippocampal interneurons to improve memory in mice (Figure 1) [30]. A positron emission tomographic study of MA stimulation at ST36 revealed elevated glucose metabolism in the left olfactory cortex and bilateral amygdaloid bodies in the MA group compared to that in a sham group in a rat model of AD generated by D-galactose injection (Table 1) [22]. Accordingly, MA at ST36 may have specific effects on regional brain activation. The regions activated by MA at ST36 were centered in the limbic system, which is involved in emotion, sensation, and memory; therefore, MA at ST36 may improve emotional processing and help patients get over fear or pain.

Acupuncture stimulation at HT7 (Sinmun) has been clinically used to treat cognitive impairment and sleep disturbances (Figure 1) [25, 31] and is considered a useful therapy for memory impairment. HT7 stimulation in a rat model of AD generated by D-galactose injection produced improvements in cognitive function and increased cortical and hippocampal glucose metabolism compared to that in control AD rats (Table 1) [32]. A shorter total reaction time in the Y maze test was observed in HT7-treated AD rats than in nontreated AD rats [32]. Similar to ST36 stimulation, MA stimulation at HT7 appears to have specific effects on cerebral glucose metabolism and thus regional brain activation in rodent models of AD.

## 3. Conclusion

In summary, data from studies on acupuncture in rodent models of AD show that MA or EA at specific acupoints improves cognitive impairment and has therapeutic effects on disease pathology. Additionally, unique acupoints appear to have targeted effects on specific neuropathological pathways. Although EA and MA are distinct techniques associated with different therapeutic benefits, they appear to induce similar effects when targeting the same acupoints in rodent models of AD. EA or MA single- or multipoint stimulation at GV20 is widely studied and is associated with effects on BDNF signaling and cognitive impairment. GV20 is located on the midline between the apices of the ears and has been a previous target for the treatment of headache in a clinical trial [33]. GV14 is another promising acupoint for managing AD, which has been studied in conjunction with EA at BL23. GV14 is located on the midline of the neck and is indicated for the treatment of memory impairment. EA stimulation at GV14 and BL23 exerts beneficial effects on pathological A*β* and tau protein generation as well as energy metabolism. Finally, single-point MA at ST36 or HT7 appears to have specific effects on regional cerebral blood flow and glucose metabolism. ST36 is located on the anterior aspect of the lower leg and it is a target for normalizing blood circulation. HT7 is located on the wrist and it is indicated for the treatment of insomnia and amnesia. Future studies should examine the exact regional brain effects of targeting these acupoints in clinical AD.

Acupuncture is a useful form of complementary/alternative medicine for the managing neurodegenerative disorders, because it can reduce the side effects of therapy as well as the financial burden of treatment on patients and their families. In addition to AD, studies have reported the utility of acupuncture treatment in Parkinson's disease, showing that it improves symptoms and has a neuroprotective role [34]. Furthermore, acupuncture has been reported as a prospective therapy for stroke [35, 36], where it could regulate glucose metabolism and be involved in poststroke neurogenesis. Acupuncture may have a potential neuroprotective role worth studying regarding the treatment of neurological diseases.

The studies summarized in this review supports the utility of acupuncture as a form of complementary medicine for AD and the ability of specific acupoint targets to address pathological disease features. Acupuncture improves cognitive impairment, decreases pathological A*β* generation, decreases neuronal apoptosis, and ameliorates neuroinflammation; however, the concept of acupoint specificity is not completely validated [4]. Moreover, the results of preclinical studies in animal models of AD require validation in clinical settings with human patients. Future preclinical and clinical studies should be expedited to inform the exact use and efficacy of different acupuncture methods and targets for the treatment of AD.

## Figures and Tables

**Table 1:** *Acupoint targets of EA and MA for the treatment of Alzheimer's disease (AD)*. GV14 and GV20 single-point EA stimulation and multipoint EA stimulation of BL23, GV26, and EX-HN3 have been preclinically studied for the treatment of AD. Single-point MA stimulation of GV20, ST36, and HT7 has also been evaluated in rodent models of AD.

	AD model	Acupoint	Frequency	Action mechanism
EA	SAMP8 [14–16]	GV14, BL23	−2 Hz, 1 mA	AMPK ↑, SIRT1, PGC1a ↓ BACE1 ↓
	A*β*1-40 injection [17, 24]	GV20, BL23	−20 Hz, 2 mA −2 Hz, 1 mA	Bcl2 ↑, BAX ↓, Notch ↓ PPAR-gamma ↑, p-p38MAPK ↓
	SAMP8 [18]	GV20, GV26, EX-HN3	−2 Hz, 0.6 mA	Glucose metabolism ↑
	APP/PS1 [20, 21, 23]	GV20	−2/15 Hz, 1 mA	NDRG2 ↓ BDNF ↑, P75 ↑, pTRK-B ↓

MA	Scopolamine injection [19]	GV20	–	BDNF ↑, CREB ↑, cholinergic system ↑
	D-Galactose injection [22]	ST36	60–90 twist/min	Glycerol metabolism ↑
	D-Galactose injection [25]	HT7	120–150 twist/min	Glucose metabolism ↑
